# The MIDWIZE conceptual framework: a midwife-led care model that fits the Swedish health care system might after contextualization, fit others

**DOI:** 10.1186/s13104-022-06198-7

**Published:** 2022-09-23

**Authors:** Helena Lindgren, Kerstin Erlandsson

**Affiliations:** 1 Department of Women’s and Children’s Health, Karolinska Institutet, Solna, Sweden; 2 Department of Health, Care and Wellbeing, Dalarna University, Falun, Sweden

**Keywords:** Midwife-led care, Conceptual framework, Evidence-based care, Multisectoral collaboration, Sub-Saharan Africa

## Abstract

**Objective:**

Familiarity with the unique tradition and experience of Swedish midwives during the more than 300 years in which midwife-led care has contributed to one of the lowest maternal and neonatal mortality and morbidity ratio in the world might encourage professionals in other countries to follow the Swedish example. The framework described below, reflecting the midwife’s role in the Swedish health care system, might, after implementation, strengthen maternal and neonatal outcomes if contextualized to other settings.

**Results:**

Using a four-step procedure we identified our topic, made a literature review, identified the key components and their internal relationship, and finally developed the MIDWIZE conceptual framework. In this framework, the midwives in collaboration with obstetricians, provide evidence-based care with continuous quality improvements during the whole reproductive life cycle. Teamwork including specialists for referral and a responsive, relational, trust-based practice is the foundation for provision of midwife-led care for healthy women with a normal pregnancy. The well-educated midwife, of high academic standard, promoting gender equality and equity is the hub in the team and the primary care provider.

## Introduction

**Fig. 1:**
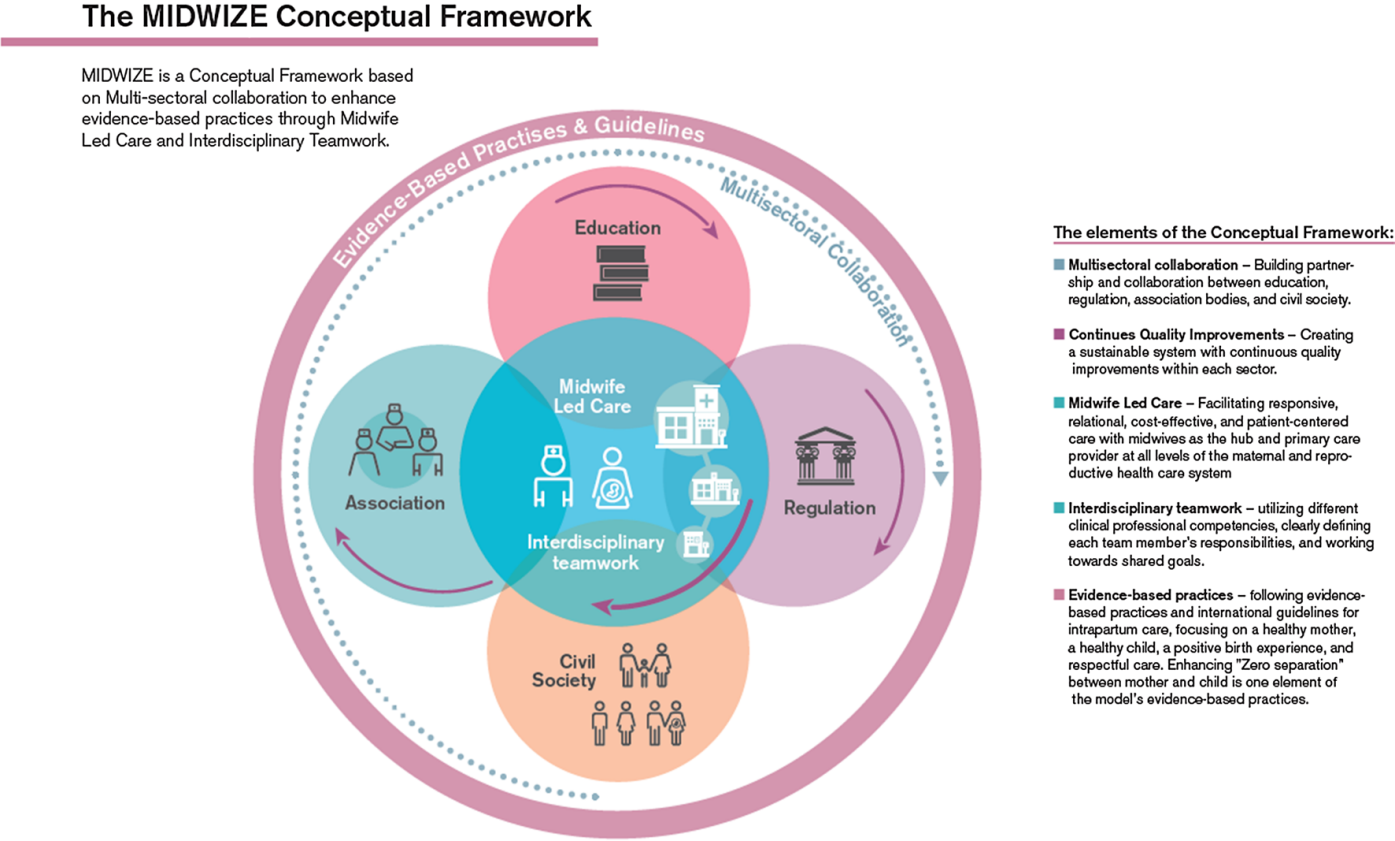
The MIDWIZE conceptual framework with it's five elements

Birth centres are defined in a Dutch Birth center study [1] as midwifery-managed locations that offer care to low-risk women during labour and birth. The Netherlands is just one of several countries where midwives have responsibility for midwife-managed birthing. Midwives work in free-standing units in some countries such as England and Germany [2] and in on-site midwife-led birth units adjunct obstetric units, in other countries [3]. Midwife-led care for low-risk women during labour and birth has been shown to be more effective with fewer interventions and better outcomes than physician-led models of care. The midwife-led model of care is further associated with decreased maternal and neonatal mortality and morbidity [4]. The State of the Worlds Midwifery report affirms that if we increase the number of midwives and the quality of care they provide, we would save an estimated 4.3 million lives a year by 2035 [5]. Planning frameworks that can guide and govern the implementation of midwife-led initiatives have been suggested by the World Bank [6]. With this background, we wish to introduce the MIDWIZE Conceptual Framework illustrating how a comprehensive midwife-led care program functions in Sweden. We provide a description of the framework to facilitate the scaling up of a system proven to be successful in the Swedish context (Fig. 1).

## Main text

### Methods

This conceptual framework was developed using the following four steps. Firstly, a topic was identified on how to explain the phenomenon [6]. Using Delphi study technique together with on-site visits to Sub-Saharan African countries (Uganda and Ethiopia), we visited governmental bodies, clinical and educational institutions. With the Swedish example as a lens, we started to identify key components of the phenomenon. Secondly, a literature review was conducted on existing studies of the topic. Using manual search in the database PubMed we identified seven studies that described the phenomenon [4, 7–12]. Thirdly, the components and their relationships were finalized based on the literature review. Finally, a framework called the MIDWIZE conceptual framework was generated to conceptualize the Swedish maternity care system. To further investigate the feasibility of implementing the MIDWIZE Conceptual Framework, we developed a 9-months training program for participants from four Sub-Saharan African countries (Ethiopia, Kenya, Malawi and Somalia) with support from the Swedish Institute [13]. In the program, the 28 participants and interdisciplinary midwife-led teams consisting of midwives, nurses, obstetricians and clinical officers at selected health care facilities in the respective countries, received training in key components of the MIDWIZE Conceptual Framework [14].

### Results

#### The MIDWIZE conceptual Framework

The term MIDWIZE is built from the words *midwife* and *wise*, illustrating the wisdom among midwives providing comprehensive care for women during their whole reproductive lifecycle. In labor and birth, this program focuses on a healthy mother, a healthy child and a positive birth experience with immediate and uninterrupted skin-to-skin care between the mother and newborn. “Zero separation”, is symbolized by the Z in the MIDWIZE framework [15]. The MIDWIZE Conceptual Framework is based on the development of the maternal health care system in Sweden in the second half of the nineteenth century [16, 17]. In parallel with this development, the midwife profession was formally regulated and given academic status. The tax-funded, free-of-charge maternal and child health care system, that was established in Sweden in the 1940’s, has resulted in almost 100% of pregnant women voluntarily utilising their right to register at the maternity services centers. Furthermore, the integration of education, association, evidence-based praxis and provision of guidelines supports the clinical midwife in her role as primary care giver for women throughout their reproductive life cycle.

#### The elements of the conceptual framework

##### Multisectoral collaboration

The idea of a multi-sectoral collaboration and interdisciplinary teamwork approach with all women primarily cared for by midwives, was considered as an approach that might save lives of mothers and their newborns. Partnership and collaboration among educational, regulatory, and professional associations were developed during the second half of the nineteenth century. This made it possible for people living in poverty and difficult social situations to improve their living conditions [17]. As a result of the long history of professionalization of midwifery in Sweden, midwives became respected as important medical professionals in the Swedish health care system.

##### Continuous quality improvements

The midwifery workforce was crucial during the second half of the twentieth century in improving both the health and dignity of mothers to be throughout the whole reproductive life cycle in parallel with positive developments in many associated fields, among them hygiene, contraception counseling, pre-conception care, abortion care, pregnancy, intrapartum and postpartum care, and care of women during and after menopause [16]. Today, midwives’ education, competence and practical skills together with a gender equality policy give the midwife greater influence in raising the quality of maternity care [18]. Continuous quality improvements were embedded throughout the twentieth century in sustainable systems within each sector of the society [17]. This in turn made it possible to develop the Swedish midwife-led care program that we now refer to as the MIDWIZE conceptual framework.

##### Midwife-led care

The midwife-led care in the MIDWIZE conceptual framework is in line with the definition of on-site midwife-led care in the international literature [1]. The main goal of introducing the MIDWIZE conceptual framework is to further explain the role of the midwife in the Swedish maternity health care system.

The midwives care for all women at the labor ward, and in uncomplicated pregnancies they act independently. In case of complications, the obstetrician takes responsibility and supports the midwife as appropriate. The midwives safeguard the promotion of physiological, spontaneous, vaginal birth. They take primary professional responsibility for the care of the women in contraception counseling, pre-conception and abortion care and care in pregnancy, intrapartum and postpartum, and care of women during and after menopause [1]. The tertiary level hospital establishment is complemented by secondary establishments e.g., local hospitals and health facilities offering more limited services. Midwives working at secondary establishments will refer women who are in need of more specialised services during the first 6 weeks post-partum and newborns during the first week of life to the tertiary level hospital for treatment and care [12].

##### Interdisciplinary teamwork

Midwives, being the professionals who best can ensure the health and wellbeing of women, girls and newborns when educated up to global standards [5] are highly respected among physicians and obstetricians in Sweden, and they are dependent on one another in the maternity health care system. It is essential that the midwives and obstetricians shift their professional responsibilities when complications occur in a responsive, relational, trust-based practice with clearly defined roles and responsibilities of each team member. The interdisciplinary team has a common goal to ensure the best outcomes and highest levels of satisfaction among women [17]. The narrower interdisciplinary teamwork between midwives and obstetricians in second or tertiary level can be broadened to include other specialists, for example, neonatologists, psychologists, physiotherapists or nutritionists when needed. At discharge from the hospital after birth, the midwife’s responsibility is to report and refer the newborn to the child health care system at the community level provided by child health or public health nurses and physicians. Facilitating cost effective and patient-centered care with midwives at the hub at all levels offers a full array of services.

##### Evidence-based practices

Following evidence-based practices and international guidelines will save lives of women and neonates and provide women and families with a positive birth experience. Zero separation between mother and child is one fundamental element of the evidence-based practices. Other examples of practices included in the MIDWIZE Conceptual Framework are, firstly, the use of dynamic birth position which allows the woman to apply a comfortable birthing position. When the woman is supported to give birth in a dynamic birth position the occurrence of perineal trauma is decreased [19] and respectful maternity care is provided. Secondly, in accordance with recent evidence, late cord clamping is a routine which is practiced by midwives in all units. These selected examples have also been implemented in health care facilities in low-income Sub-Saharan African countries [20].

### Discussion

The drive and desire to advance Swedish societal development since the 1930s has led to extending the scope of practice for midwives, starting with the establishment of free maternity care. Contraceptive counseling and the midwife’s work with public health developed during the same period and later midwives began to provide quality abortion care. There is today a linkage between the UN Agenda 2030 and the midwife’s role and scope of practice, and to protect midwives’ rights, including the academisation of the profession and decent work load in practice environments [17]. Sweden was one of the first countries in the world to have midwives’ salaries placed on the government’s agenda 100 years ago, fulfilling global standards for midwifery education and practice before such standards existed. However, Sweden together with many other countries, still needs to invest in developing midwifery leadership further to accelerate the implementation of the 2030 Agenda [17]. Midwife-led care located on-site at obstetric units has been a significant success in establishing Sweden as one among the 5 countries with the best maternal and neonatal outcomes in the world.

The MIDWIZE conceptual framework, might be particularly important to establish in low and middle-income countries where large numbers of maternal and neonatal deaths occur in secondary level health facilities. Replication of the framework contextualized and implemented in other settings might improve poor maternal and newborn outcomes and ensure access to safe quality care [1]. The MIDWIZE conceptual framework could advocate for midwives as a driving force for change in other settings than the Swedish one. The framework has been piloted with good results in Kenya, Malawi and Ethiopia and will now be tested in a research project in Uganda for strength and weaknesses in a low resource setting unlike the Swedish one. The study is registered at ClinicalTrials.gov (NCT05237375).

### Limitations

- Already existing health care structures differ, and are shaped by values, beliefs, and experience, thus each setting require different actions to establish midwife-led care in line with the MIDWIZE conceptual framework.
- Contextualization can be the first step prior to the introduction of the MDWIZE conceptual framework in already existing health care structures.

## Data Availability

Not applicable.
